# Predictors of Participant Retention in a Guided Online Self-Help Program for University Students: Prospective Cohort Study

**DOI:** 10.2196/jmir.2323

**Published:** 2013-05-22

**Authors:** Magdalena Wojtowicz, Victor Day, Patrick J McGrath

**Affiliations:** ^1^ Department of Psychology and Neuroscience Dalhousie University Halifax, NS Canada; ^2^ Dalhousie Counselling Centre Dalhousie University Halifax, NS Canada; ^3^ Department of Psychology and Neuroscience, Psychiatry, & Pediatrics Dalhousie University Halifax, NS Canada; ^4^ IWK Health Centre Halifax, NS Canada

**Keywords:** self-help, online treatment, Web-based, retention, dropouts, theory of planned behavior

## Abstract

**Background:**

Attrition is a persistent issue in online self-help programs, but limited research is available on reasons for attrition or successful methods for improving participant retention. One potential approach to understanding attrition and retention in such programs is to examine person-related variables (eg, beliefs and attitudes) that influence behavior. Theoretical models, such as the Theory of Planned Behavior, that describe conditions influencing human behavior may provide a useful framework for predicting participant retention in online-based program.

**Objective:**

We examined predictors of participant retention in a guided online anxiety, depression, and stress self-help program for university students using the theory of planned behavior. We also explored whether age, symptom severity, and type of coaching (ie, email vs phone) affected participant retention.

**Methods:**

65 university students with mild to moderate depression, anxiety, and stress were enrolled in this prospective cohort study. Participants completed a questionnaire based on the theory of planned behavior prior to commencing the online-based program and the Depression Anxiety and Stress Scale (DASS) during the assessment module of the program. Participant retention was operationalized as the number of program modules completed.

**Results:**

Perceived control over completing the online program significantly predicted intention to complete the program (*F*_3,62_=6.7; *P*=.001; adjusted *R*^2^=.2; standardized beta=.436, *P*=.001). Age (standardized beta=.319, *P*=.03) and perceived behavioral control (standardized beta=.295, *P*=.05) predicted the number of program modules completed (*F*_3,61_=3.20, *P*=.03, adjusted *R*^2^ =.11). Initial level of distress (ie, symptom severity) did not predict participant retention (*P*=.55). Participants who chose phone-based coaching completed more program modules than participants who chose email-based coaching (Mann-Whitney’s *U*=137; *P*=.004).

**Conclusions:**

Participants’ age, level of perceived behavioral control, and choice of interaction (ie, phone-based or email-based coaching) were found to influence retention in this online-based program.

## Introduction

Online self-help programs designed to assist individuals with mental health concerns have been demonstrated to be effective at improving mental health outcomes [1,2]. Despite the noted efficacy of such programs, participant dropout is a persistent issue, with reported attrition rates reaching between 50-83% [3-5]. Few studies have formally investigated factors contributing to participant attrition from Internet-based programs, yet such research would provide crucial information towards improving participant retention and, therefore, improve mental health outcomes.

There is currently little known about successful approaches for improving retention in online programs. Studies using guided Internet programs (eg, coach-assisted) have reported relatively lower dropout rates compared to unguided programs (eg, [6]). However, direct comparisons of retention in guided and unguided programs are currently not available. One recent pilot study evaluated whether the addition of a program coach would increase participation in an online program for promoting self-management in bipolar disorder [7]. The authors found that participants who were given an online coach were more likely to return to the program after registration (71% initial retention) and continue in the program after 3 weeks (38% retention) compared to participants without a program coach (44% and 9% retention, respectively) [7]. Some studies have also investigated the use of phone support in improving participant retention. Kenwright and colleagues found that participants who received phone support during a computer-guided self-help program for obsessive-compulsive disorder were less likely to drop out than those who did not receive phone support [8]. In contrast, Anderson and colleagues did not observe a decrease in dropout rates from participants receiving weekly telephone calls compared to those who received only the online-based program [9]. However, some research suggests that receiving reminders, regardless of the type (eg, phone, in person, postcard), has a positive effect on increasing retention [10,11].

There is limited research available on predictors of attrition from online programs. The few studies examining this issue have focused primarily on sociodemographic factors (eg, age, sex, years of education), disorder characteristics (eg, symptom severity and duration), and/or treatment-related variables (eg, treatment setting) [4,12-15]. To date, discrepant relationships have been reported between all of these variables and attrition, with the exception of the symptom severity [4]. Studies exploring the influence of symptom severity on dropout rates have found that individuals with less severe difficulties were more likely to drop out of online-based programs [12-14]. These findings suggest that individuals with less severe distress may be either less motivated or may benefit less from treatment and, therefore, be more likely to drop out. Although this research provides some insight into reasons for participant dropout, further research is necessary to better understand factors contributing to participant attrition and retention.

An alternative approach to understanding attrition is to examine person-related factors (eg, beliefs, attitudes) that influence the behavior. One theory that attempts to identify predictors of behavior is the Theory of Planned Behavior [16,17]. This theory stipulates a person’s (1) attitudes towards the behavior, (2) subjective normative beliefs (ie, perception of what other people think about the behavior and how much they are influenced by others’ beliefs), and (3) perceived behavioral control (ie, how much control they believe they have over completing the behavior) drive the person’s intention to complete the behavior. Intention and perceived behavioral control, in turn, predict the likelihood the person will engage in the behavior. The theory of planned behavior has been used extensively to investigate and predict changes in health behaviors, including increasing physical activity, reducing risk-taking behavior (eg, speeding, drinking alcohol, using drugs), and dietary changes [18,19]. This theory may provide a useful framework for investigating person-related factors that influence retention in online-based self-help programs.

The present study aimed to investigate predictors of participant retention using the theory of planned behavior. We prospectively examined how participants’ attitudes, subjective normative beliefs, and perceived behavior control predict intention to complete a guided online anxiety, depression, and stress self-help program for university students. We then examined whether intention and perceived behavior control successfully predict participant retention in the program. Finally, given the literature examining sociodemographic, disorder characteristics, and treatment-related variables on attrition, we explored whether age, symptom severity, and type of coaching (ie, email vs phone) affected participant retention.

## Methods

### Participants and Recruitment

Participants were recruited from Dalhousie University, the University of King’s College, and Nova Scotia College of Art and Design, in Halifax, Canada. Recruitment for the study began in 2010. Participants were recruited primarily via emails, advertisements in a campus newspaper, and recruitment posters. Interested individuals contacted the primary program coach through email and were provided with information regarding the study. The study protocol was initially presented to participants on the website’s consent form page. Interested participants signed up for the program online and received a phone call from the primary program coach, who reviewed the consent form with the participant and assessed their eligibility for the study. Participants who provided verbal and written informed consented and met study criteria were included in the study.

**Figure 1:**
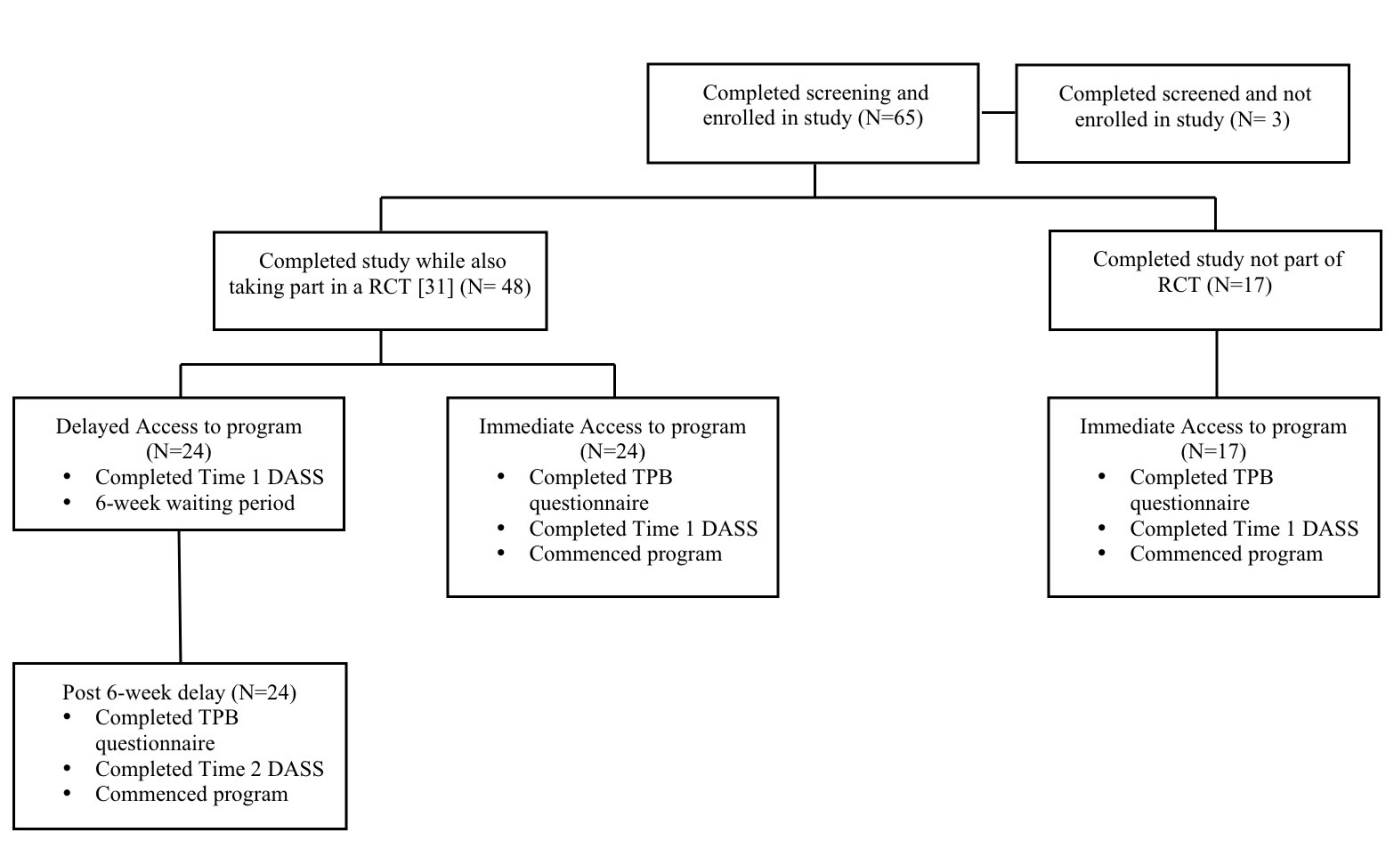
Flowchart of participant recruitment.

All participants provided informed consent following procedures approved by the Dalhousie University Research Ethics Board. Eligible participants met the following criteria: (1) experiencing mild to moderate levels of anxiety, depression, or stress, (2) not experiencing suicidal thoughts, (3) not experiencing symptoms of bipolar disorder, an eating disorder, an addiction, or psychosis, and (4) not receiving psychological counseling. Eligibility for the study was assessed through a phone conversation with the participant and the program coach. In total, 68 university students attending the aforementioned postsecondary institutes were assessed for eligibility. Three were excluded due to not meeting eligibility criteria (ie, decided to seek personal counseling), and 65 were enrolled to participate in the study. From this sample, 48 subjects were also participants in a controlled clinical trial of the online self-help program, and for this reason, 24 participants experienced a 6-week delay in accessing the program due to being randomly assigned to a delayed access control condition. However, all participants, regardless of assignment, completed all measures (ie, the Theory of Planned Behavior Questionnaire and Depression Anxiety Stress Scale-21) immediately prior to commencing the program (see Figure 1).

### Measures

#### Theory of Planned Behavior Questionnaire

The Theory of Planned Behavior Questionnaire (see Multimedia Appendix 1) is a brief 10-item questionnaire designed to quantitatively measure participants’ attitudes, subjective normative beliefs, perceived behavioral control, and intention to complete the guided online self-help program. This questionnaire was developed using guidelines outlined by Fishbein & Ajzen [20] and Francis and colleagues [21].

#### Depression Anxiety Stress Scale-21 (DASS-21)

The DASS-21 [22] is an abbreviated version of the original 42-item DASS. It is composed of three 7-item subscales measuring symptoms of depression, anxiety, and stress. Participants are asked to rate the degree to which they endorse each item on a 4-point Likert scale. The DASS-21 has been found to be a valid measure of depression, anxiety, and stress [23].

### Study Design

All participants who met eligibility criteria and provided written consent were asked to complete the Theory of Planned Behavior Questionnaire via email prior to commencing the guided online self-help program. For participants who received immediate access to the program, the questionnaire was collected immediately prior to beginning the program modules. Participants who were given delayed access to the program, as part of the randomized control trial, completed the questionnaire 6-weeks post assignment (ie, immediately prior to beginning the program modules). Hence all participants completed the questionnaire immediately prior to commencing the program. The DASS-21 was completed as part of the Introduction and Assessment Module. Delayed access participants completed the DASS-21 for a second time (6-weeks post assignment) as part of the first module (ie, Introduction and Assessment Module).

#### Online Self-Help Program

**Figure 2:**
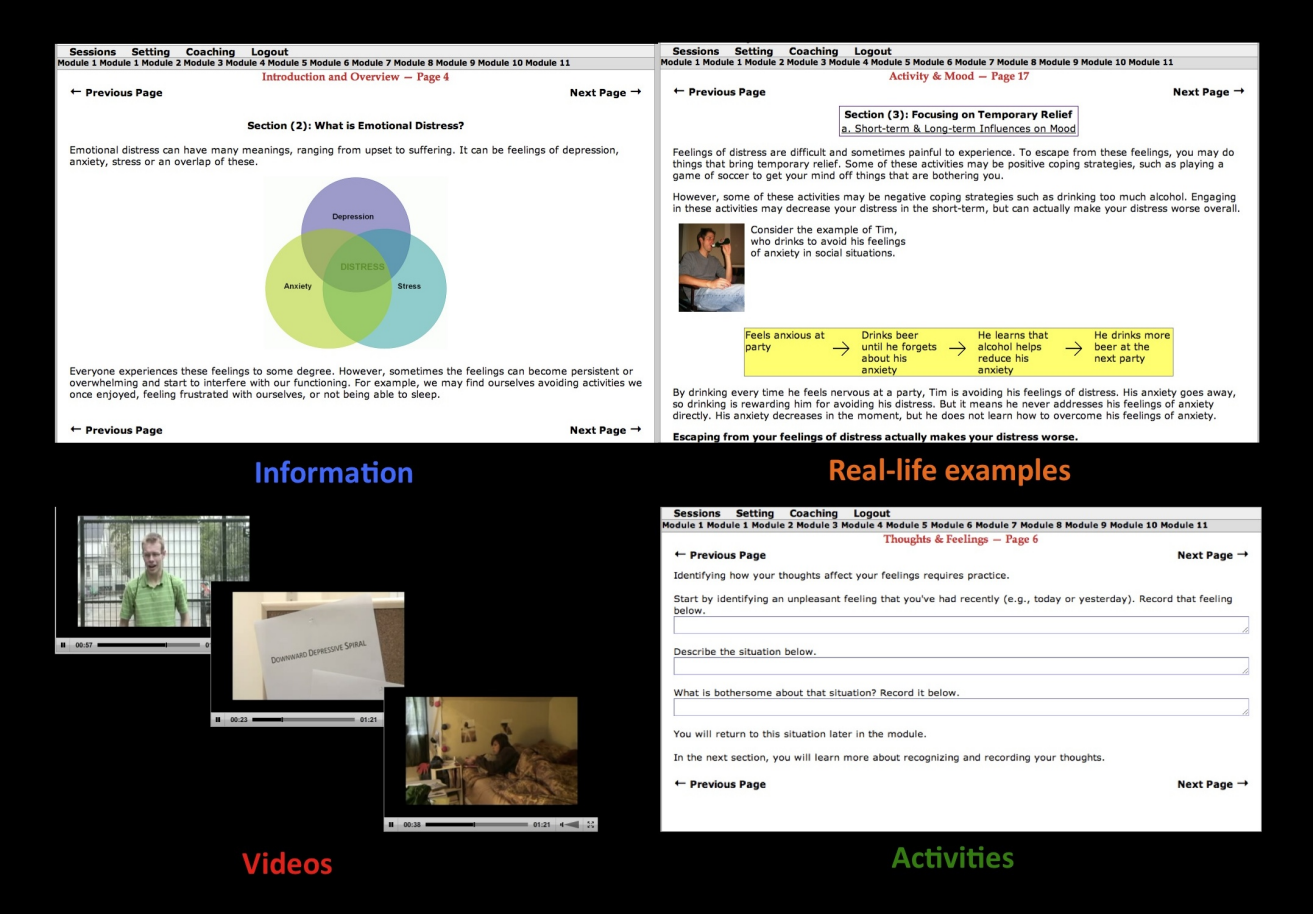
Screenshots of the online-based program.

This is a cognitive-behavior therapy-oriented online self-help program developed at Dalhousie University to assist students experiencing mild to moderate anxiety, depression, and stress. It consists of 5 core modules (Introduction and Assessment, Activity and Mood, Motivation, Thoughts and Feelings, and Advanced Thoughts and Feelings; see Table 1 for description of each module). Each module is organized in a workbook format that includes psychoeducation, real-life examples, videos, and activities (see Figure 2). This program has been demonstrated to reduce self-reported symptoms in depression, anxiety, and stress in participants who complete the program compared with wait-list controls [24].

#### Program Coaches

Participants were assigned to a program coach (ie, a trained graduate or undergraduate student), who contacted them via telephone or email (based on the participants’ preference) on a weekly basis. On average, contact with a coach involved one phone call or one email a week. Phone calls were on average 15-20 minutes in duration, while emails were on average 250-500 words in length. Participants were informed during the consent process that the program coaches were not professional counselors. Program coaches did not function as individual therapists; rather, they provided motivational support to participants and clarified information contained within the program. Coaches also monitored participant progress and reviewed activities completed by participants to ensure the correct applications of the techniques.

Program coaches were trained on the online self-help program by the primary investigator, Dr. Victor Day, as well as the head program coach. The majority of the training involved reviewing the online program materials in order to adequately address participant questions and provide assistance with module activities, when necessary. Program coaches were supervised by the head program coach and the primary investigator in order to assure fidelity to the coaching model.

### Data Analysis

The data were analyzed using the statistical software package SPSS 17.0. A planned multiple regression was conducted to investigate whether participants’ attitudes, subjective normative beliefs, and perceived behavioral control predicted their intention to complete the online-based program. A planned hierarchical regression was conducted to investigate whether intention, perceived behavioral control, and age predicted participant retention (ie, the number of modules completed). These analyses were conducted in accordance to the methods utilized by Azjen [16,17], Fishbein & Ajzen [20], and those outlined by Francis and colleagues [21]. Nonparametric Mann-Whitney tests were used to investigate differences between immediate and delayed access participants as well as participants who chose phone-based or email-based coach contact.

**Table 1:** Description of core modules.

Core modules	Topics	Exercises
1. Introduction	Introduction to program features. Description of emotional distress.	Depression, Anxiety, and Stress Questionnaire. Suicidal Ideation screener.
2. Activity and Mood	Relationship between activity and mood.	Identifying goals for change. Decisional balancing chart.
3. Motivation	Building motivation for change.	Identifying steps to achieve goals. Identifying and planning for barriers to change.
4. Thoughts and Feelings	How thoughts affect feelings. Identifying and challenging thoughts.	Thought records. Labeling common cognitive distortions. Challenging negative thoughts.
5. Advanced Thoughts and Feelings	Challenging more persistent thoughts and core beliefs.	More practice of challenging thoughts. Identifying automatic thoughts and core beliefs. Challenging automatic thoughts and core beliefs. Depression, Anxiety, and Stress Questionnaire.

## Results

### Participant Characteristics

See Table 2 for a summary of participant characteristics. All participants endorsed moderate levels of depression, anxiety, and stress prior to commencing the guided online self-help program.

### Delayed Access Versus Immediate Access

A proportion of the sample (24/65, 37%) completed the questionnaires and online program 6 weeks after enrolling into the study; thus, the potential effect of delayed access on participant retention was examined using a nonparametric Mann-Whitney test. Participants who experienced delayed access completed a similar number of program modules (mean 2.79, SD 1.44; median 2.5). This was compared to participants who received immediate access (mean 4.33, SD 3.22; median 3.0; *U*=439; *P*=.46). A larger proportion of delayed access participants discontinued the program (ie, did not complete all 5 modules; 19/24, 80%) compared to immediate access participants (23/41, 56%). However, this difference was found to be only marginally significant when examined using a chi-square test (χ^2^_1_=3.38; *P*=.068; N=65). When comparing only the subjects who were also enrolled in the concurrent randomized controlled clinical trial, 62.5% (15/24) of immediate access subjects completed all 5 modules, while 20.8% (5/24) of the delayed access subjects completed all 5 modules (χ^2^_1_=8.57; *P*=.003; N=48).

### Factors Predicting Intention

To examine whether attitudes, subjective normative beliefs, and perceived behavioral control predict participants’ intention to complete the guided online-based program, these variables were entered into a simultaneous multiple regression (see Table 3). The model was significant (*F*_3,62_=6.7; *P*=.001; adjusted *R*^2^ =.21), however, only perceived behavioral control was found to significantly predict participants’ intention to complete the guided online self-help program (standardized beta=.436, *P*=.001). Thus, participants who endorsed higher perceived behavioral control over completing the online program also endorsed higher intention to complete the program.

**Table 2:** Description of participant characteristics.

Participant characteristics	Total N=65
Age, mean (SD)	23.2 (5.0)
Males (Females)	9 (56)
Dropout^a^, n	42
Modules completed, mean (SD)	3 (1.6)
Initial Depression Score, mean (SD)	19.4 (11.7)
Initial Anxiety Score, mean (SD)	12.5 (9.0)
Initial Stress Score, mean (SD)	21.5 (8.9)

^a^Dropout was defined as an individual who did not complete all 5 modules.

**Table 3:** Multiple regression predicting intention to complete the online-based program.

	B	SE^a^ B	Standardized beta
Attitude	.072	.078	.126
Subjective norms	-.013	.013	-.128
Perceived behavioral control	.325	.090	.436^b^

^a^SE=standard error.

^b^*P*=.001

### Factors Predicting Program Completion

A hierarchical multiple regression was employed to examine factors that may affect participant retention, defined as the number of program modules completed. Intention and perceived behavioral control were entered in the first model since these two variables are proposed to predict behavior (ie, the number of modules completed) according to the theory of planned behavior [17]. Age was then entered into the second model because previous literature on attrition from online-based programs has found that age can influence program adherence [12,15,25]. Model 1 was not found to significantly predict the number of modules completed (Model 1: *F*_2,62_=1.29, *P*=.28 adjusted *R*^2^ =.01; see Table 4). Rather, the best fitting model for predicting participant retention was the combination of perceived behavioral control, intention, and age (Model 2: *F*_3,61_=3.20, *P*=.03, delta *R*^2^ =.10). In this combined model, perceived behavioral control (standardized beta=.295, *P*=.038) and age (standardized beta=.319, *P*=.012) significantly predicted the number of modules completed (see Table 4). A follow-up analysis was conducted to examine whether moderation was occurring between perceived behavior control and age. The moderation analysis was nonsignificant (*P*=.78), suggesting that the presence of age did not moderate the relationship between perceived behavioral control and participant retention. Instead, these two variables in conjunction predict participant retention.

**Table 4:** Hierarchical multiple regression predicting retention (ie, the number of modules completed) in the online-based program.

		B	SE^a^ B	Standardized beta
**Model 1**				
	Intention	-.058	.214	-.038
	Perceived behavioral control	.242	.160	.215
**Model 2**				
	Intention	-.182	.210	-.120
	Perceived behavioral control	.332	.157	.295^b^
	Age	.105	.040	.319^c^

^a^SE=standard error.

^b^*P*=.038.

^c^*P*=.012.

### Initial Distress

Initial level of distress (ie, severity of depression, anxiety, and stress symptoms), as measured by DASS scores, did not predict the number of modules completed: *F*_3,61_=7.14, *P*=.55, adjusted *R*^2^ =-.01.

### Phone-Based Versus Email-Based Coaching

The effect of the type of contact with the program coach on participant retention was examined using a Mann-Whitney test because of unequal group *N* s; 83% (54/65) of participants chose primarily email-based contact with the program coach. Participants who chose phone-based coaching completed more program modules (mean 4.36, SD 1.12; median 5.0). This was compared to participants who chose email-based coaching (mean 2.81, SD 1.61; median 2.0; *U*=137; *P*=.004). Participants who chose phone-based coaching did not differ from participants who chose email-based contact on any of the theory of planned behavior variables (ie, attitudes, subjective normative beliefs, perceived behavioral control, and intention) or on DASS scores, as examined by a series of Mann-Whitney tests (*P*>.05).

## Discussion

The purpose of this study was to investigate predictors of participant retention in a guided online program for anxiety, depression, and stress in university students through the use of the theory of planned behavior. Within our sample, 65 % (42/65) of participants did not complete all 5 core modules of the program. Although this represents a large proportion of noncompleters, the percentage of dropouts fell within previously reported ranges [3,4] and may have been, in part, influenced by the fact that some participants were also enrolled in a randomized control trial of the program [5]. Overall, attrition rates have been found to be lower in randomized control trials than in open access Web-based studies, with lower completion rates for individuals in the experimental intervention group than those in the control group [3,5]. In our study, we found that participants who were given delayed access completed a similar number of program modules compared with those who were given immediate access. The proportion of participants who did not complete all of the modules appeared to be larger in delayed access participants (19/24, 80%) than immediate access participants (23/41, 56%). However, this difference was only marginally significant (*P*=.068) and was likely influenced by an unequal number of individuals who experienced delayed access versus immediate access (24 versus 44, respectively). To examine this further, we compared the completion rates of only the subjects who were concurrently enrolled in the randomized controlled trial (N=48). We found that, in this instance, a greater proportion of immediate access participants (15/24, 62.5%) completed all 5 program modules compared to delayed access participants (5/24, 4.17%; *P*<.01). The result of this subanalysis contradicts previous findings of lower completion rates for individuals in experimental conditions compared with control conditions (eg, [3]) and suggests that delayed access in randomized controlled trials may negatively affect participant retention.

According to the theory of planned behavior [16,17], a person’s attitudes, subjective normative beliefs, and perceived behavioral control influence their intention to engage in a behavior. We examined how these variables influenced participants’ intention to complete the online self-help program. We found that the participants’ perceived control over completing the online self-help program significantly predicted self-reported intention to complete the program. Thus, university students who endorsed greater perceived control over their behavior also endorsed greater intention to complete the online program. This finding is consistent with previously reported research on health behaviors, which has shown that perceived behavioral control is an important predictor of behavioral intention as well as actual behavioral change [19,26].

Ajzen [17] asserts that intention to complete a behavior and perceived control over the behavior predict whether a person will engage in the behavior. We did not find that these two variables alone (ie, intention and perceived behavioral control) significantly predicted the number of modules completed. Instead, we found that age and perceived behavioral control significantly predicted participant retention. Older university students, who endorsed more perceived control over completing the online-based program, actually completed more program modules. Previous studies have found that younger participants are more likely to drop out than older participants, though these studies included adults with a broad range of ages [12,15,25]. Our study found that even in young adulthood (ages 19-28), relatively older participants were more likely to complete online self-help programs. As mentioned above, we also found that when age was entered into the model, greater perceived behavioral control was also found to predict the number of modules completed. The concept of perceived behavior control is closely related to self-efficacy (ie, a person’s judgment of how successfully they can complete a behavior) [17]. Our findings are consistent with health psychology research demonstrating that those with high perceived behavioral control (ie, high self-efficacy) are more likely to adhere to exercise programs [27,28]. Promoting self-efficacy within this target group (ie, university students) may help improve participant retention. Suggested methods for improving self-efficacy include motivation interviewing [29], focusing on the students’ previous successes, vicarious experience (ie, the successes and failures of their peers), and verbal praise [30,31]. These strategies have been demonstrated to improve academic self-efficacy and performance in university students [32,33]. Identifying participants with low self-efficacy and attempting to foster this self-efficacy through the aforementioned strategies may potentially improve adherence and retention in online-based programs for this population. For example, this could be done during an initial phone conversation by reviewing the costs and benefits of changing the participant’s current behavior, discussing when participants can work on the program on a weekly basis, examining past successful life changes, and praising module completion in weekly email or phone conversations. Further research in this area is warranted.

Of note is the absence of a predictive relationship between participants’ intention to complete the online program and the number of modules completed. Consistent with this finding, a recent study investigating attrition from an online treatment for chronic insomnia also failed to find a relationship between intention to complete the program and attrition [26]. The lack of a relationship between intention and behavior may be affected by the study design. Few studies using the theory of planned behavior include prospective designs or objectively measure behavior. When behavior is measured, it is generally done through self-report [18,19]. Within the theory of planned behavior literature, prediction of observable behavior has been found to be more modest than prediction of self-reported behavior [18]. The lack of relationship between self-reported intention and behavior in our study may, in part, be due to the fact that we employed a prospective design and objectively measured behavior. Our relatively modest sample size may have also contributed to the absence of this relationship.

Previous literature on attrition from online-based programs has examined sociodemographic, symptom severity, and treatment characteristics. Some studies have found consistent relationships between the degree of symptom severity and participant dropout [4]. In particular, participants with less severe symptoms have been found to be more likely to drop out from online programs for chronic insomnia, posttraumatic stress, phobia, and panic disorders [12-14,26]. In our study, we did not find that severity of depression, anxiety, and/or stress predicted participant retention. However, unlike many of the aforementioned studies, participants in our study were not required to meet diagnostic criteria for a psychological disorder. Rather, the majority of our sample included university students with subclinical levels of anxiety, depression, and stress. The absence of a predictive relationship between symptom severity and participant retention may have been due to our subclinical sample.

Guided Internet programs for anxiety and depression have been found to be more efficacious than interventions without treatment support [34]. These benefits are obtained even when the individuals providing treatment support are not professionally trained therapists [35,36]. Our program included nontherapist coaches who provided encouragement and support to participants via telephone or email, based on participant preference. The majority of participants chose primarily email-based contact (83%). Yet, participants who chose phone-based coaching completed significantly more modules (on average 4.36 modules) than students who chose email-based coaching (on average 2.8 modules). Given that participants were provided with the option to receive phone- or email-based coaching, this finding might represent a self-selection bias (ie, participants who were more likely to complete the program selected to receive phone-based coaching). However, Kenwright and colleagues found that participants who were randomized to receive scheduled clinician-directed phone calls were less likely to drop out from a computer-aided intervention for obsessive compulsive disorder [8]. Phone-based support may facilitate engagement and rapport, enhancing the participants’ experience in the online-based program, thus improving participant retention. Future research should examine the utility of phone-support as a strategy to improve participant retention in online-based programs.

### Limitations

Our study utilized a questionnaire based on the theory of planned behavior, which was designed to be brief and to address our specific hypotheses (ie, predicting participant retention in the online-based program). Further research is necessary to establish the reliability and internal validity of the items in this questionnaire. In addition, the university students who enrolled in the study were primarily female, and hence gender differences in participant retention could not be examined.

### Conclusions

This study contributed to the limited literature on predictors of participant attrition and retention in online-based programs. Age, perceived control over completing the program, and the type of coach contact (ie, phone) were found to be important factors in increasing participant retention. Further research is necessary to examine the potential influence of phone-based support versus email support on participant retention. Interventions exploring potential approaches for fostering participants’ perceived behavioral control (ie, self-efficacy) may also be helpful in developing strategies to improve participant retention in online programs.

## Multimedia Appendix 1

Theory of planned behavior questionnaire.
