# Supplementary material for: Predictors of Participant Retention in a Guided Online Self-Help Program for University Students: Prospective Cohort Study
Source: J Med Internet Res. 2013 May 22;15(5):e96. doi: 10.2196/jmir.2323 (PMC3668607; doi:10.2196/jmir.2323)
Supplement: Supplementary file 1 [file jmir_v15i5e96_app1.pdf]

Theory of Planned Behavior Questionnaire

Instructions:
Please make sure to read the questions and response options carefully. Place an X next to the response that best describes your opinion.

1. I intend to complete this online-based program in the next 5-6 weeks
( ) Extremely agree
( ) Agree
( ) Slightly agree
( ) Neither agree nor disagree
( ) Slightly disagree
( ) Disagree
( ) Extremely disagree

2. For me, completing this online based-program over the next 5-6 weeks is:
( ) Extremely worthless
( ) Worthless
( ) Slightly worthless
( ) Neither worthless nor valuable
( ) Slightly valuable
( ) Valuable
( ) Extremely valuable

3. I think that, completing this online based-program over the next 5-6 weeks will reduce the amount of anxiety/depression/stress that I feel:
( ) Extremely likely
( ) Likely
( ) Slightly likely
( ) Neither likely nor unlikely
( ) Slightly unlikely
( ) Unlikely
( ) Extremely unlikely

4. Reducing the amount of anxiety/depression/stress that I feel is:
( ) Extremely important
( ) Important
( ) Slightly important
( ) Neither important nor unimportant
( ) Slightly unimportant
( ) Unimportant
( ) Extremely unimportant

5. The important people in my life would want me to complete a self-help program like this online based-program.
( ) Extremely agree

( ) Agree
( ) Slightly agree
( ) Neither agree nor disagree
( ) Slightly disagree
( ) Disagree
( ) Extremely disagree

6. When it comes to addressing your feelings of anxiety/depression/stress, how much are you guided by the opinion of important people in your life?
( ) Not at all guided
( ) Unguided
( ) Slightly unguided
( ) Neither guided nor unguided
( ) Slightly guided
( ) Guided
( ) Extremely guided

7. For me, completing this online based-program in the next 5-6 weeks will be:
( ) Completely impossible
( ) Impossible
( ) Slightly impossible
( ) Neither possible nor impossible
( ) Slightly possible
( ) Possible
( ) Completely possible

8. How much control do you believe you have over completing this online based-program over the next 5-6 weeks?
( ) Absolutely no control
( ) No control
( ) Slightly no control
( ) Neither control nor no control
( ) Some control
( ) Control
( ) Complete control

Think of an external factor that would make it difficult for you to complete this online based-program in the next 5-6 weeks (e.g. school, work, busy schedule, etc).

PLEASE ENTER THAT FACTOR HERE:

9. In the next 5-6 weeks I expect that this factor will be:
( ) Extremely demanding
( ) Demanding
( ) Slightly demanding
( ) Neither demanding nor undemanding

( ) Slightly undemanding
( ) Undemanding
( ) Extremely undemanding

10. In the next 5-6 weeks how do you expect the demands of this factor will affect your ability to complete this online based-program:
( ) Extremely difficult
( ) Difficult
( ) Slightly difficult
( ) Neither difficult nor easy
( ) Slightly easy
( ) Easy
( ) Extremely easy
